# Mixed infection of three nontuberculous mycobacteria species identified by metagenomic next-generation sequencing in a patient with peritoneal dialysis-associated peritonitis: a rare case report and literature review

**DOI:** 10.1186/s12882-023-03156-8

**Published:** 2023-04-13

**Authors:** Xiangfeng Chen, Jie Zhu, Zhou Liu, Jun Ye, Liqi Yang, Zhenhua Zhang

**Affiliations:** Department of Infectious Diseases, The Second Affiliated Hospital of Anhui Medical University, Hefei, China

**Keywords:** Peritoneal dialysis, Peritonitis, Nontuberculous mycobacteria, Mixed infections, Case report

## Abstract

**Background:**

Peritonitis caused by nontuberculous mycobacteria (NTM) is an infrequent but important complication in patients undergoing peritoneal dialysis (PD). There has been no report of mixed infections with multiple NTM. Peritoneal dialysis-associated peritonitis (PDAP) caused by *Mycobacterium abscessus* is more common than that caused by *M. smegmatis* and *M. goodii*.

**Case presentation:**

This case concerns a patient with PDAP caused by gram-positive bacilli, which could not be identified at the species level in successive detections of initial peritoneal effluent. Later, *M. smegmatis* was detected with no sensitivity results in bacterial culture. However, metagenomic next-generation sequencing (mNGS) and first whole-genome sequences indicated that there were three species coexisting in the culture, including *M. smegmatis* (24,708 reads), *M. abscessus* (9224 reads), and *M. goodii* (8305 reads). This is the first case of PDAP with specific evidence that conventional detection methods isolated a poorly pathogenic NTM, whereas mNGS and first whole-genome sequences identified multiple NTM. Pathogenic bacteria might not be detected using conventional methods due to their lower abundance. This case report is the first description of mixed infections with more than two species of NTM during PDAP.

**Conclusions:**

PDAP caused by multiple NTM is rare, and the diagnosis is difficult. When NTM are isolated by conventional tests in patients who are suspected of infection, clinicians should be vigilant, and further tests should be performed to determine the presence of rare or even previously unknown bacteria, for which the quantity is relatively low, but the pathogenicity is high. The rare pathogen may be a primary agent in causing such complications.

**Supplementary Information:**

The online version contains supplementary material available at 10.1186/s12882-023-03156-8.

## Background

Peritoneal dialysis-associated peritonitis (PDAP) is a common complication of peritoneal dialysis (PD), including peritonitis according to organisms identified on culture, culture-negative peritonitis, catheter-related peritonitis (either exit-site or tunnel), and enteric peritonitis, which are caused by single pathogens in most cases, with only 4.8–16.0% being poly-bacterial infections [1, 2]. These infections are associated with higher rates of relapse, hospitalization, catheter removal, permanent conversion to hemodialysis, and even death, compared to those with single-pathogen peritonitis [3].

The incidence of pure nontuberculous mycobacteria (NTM) in PDAP was approximately 0.3% [4], and although relatively rare, related case reports have been gradually increasing, and their poor prognosis has attracted the attention of clinicians [5]. The top three species causing PDAP are *Mycobacterium fortuitum*, *M. chelonae*, and *M. abscessus* [6], and *M. abscessus* was classified as a *M. chelonae* subspecies prior to 1992 [7]. Thus, its previous incidence could have been underestimated. There have been only three case reports regarding the single-pathogen *M. smegmatis* complex in PDAP [8–10], two of which were identified as *M. wolinskyi*, one as *M. smegmatis*, and none as *M. goodii.*

The current research papers regarding poly-microbial PDAP are often classified without separating NTM because of their low incidence [11]. However, mixed infections associated with NTM at other sites, especially in the lungs, often coexist with *M. tuberculosis* (MTB), with rates ranging from 1.4 to 7.1% [12, 13]. Studies on lung infections caused by NTM have shown the incidence of multiple NTM co-infections fluctuating from 8 to 61% [14–17], with most being a *M. avium* complex (MAC) with the coexistence of a *M. abscessus* complex (MABC). Identification of multiple NTM in the same specimen at other sites presented only in two case reports. One involved pleural effusion caused by *M. fortuitum* and *M. mageritense* [18] and the other involved a brain abscess caused by *M. immunogenum* and *M. llatzerense* [19]. Because neither was focused on PDAP, we present the first case regarding a patient with PDAP, for which *M. smegmatis* was isolated in a conventional culture, whereas three species of NTM were identified by mNGS and first whole-genome sequences, suggesting that the causative pathogen might not have been *M. smegmatis.*

## Case presentation

**Fig. 1:**
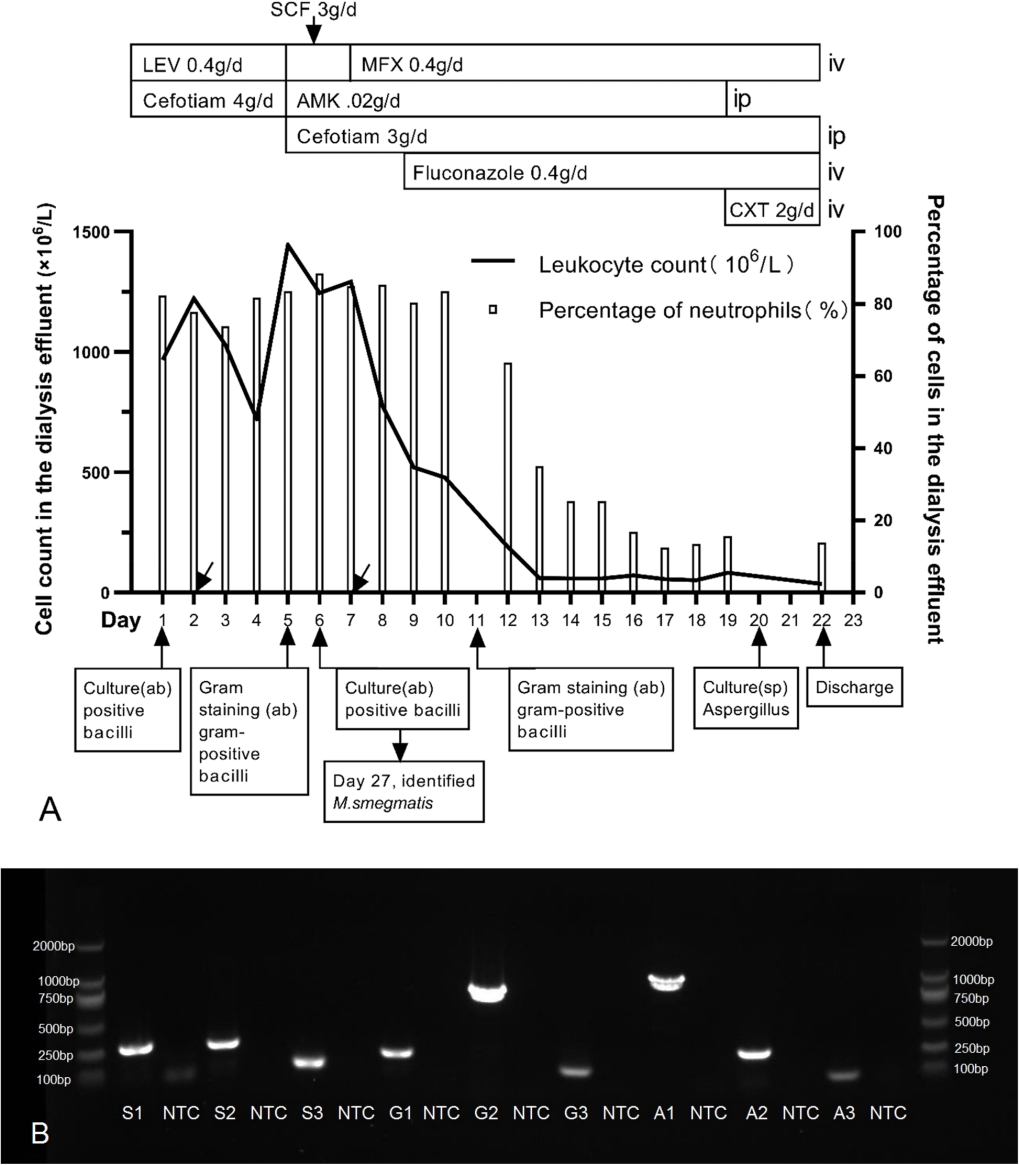
The diagnosis and treatment process of the patient and etiology testing. **a** Clinical course of the patient. LEV: levofloxacin; SCF: cefoperazone sulbactam; MFX: moxifloxacin; AMK: amikacin; CXT: cefoxitin; ab: abdominal dropsy; sp.: sputum; iv: intravenous; ip: intraperitoneal; ↙ means inject vancomycin 2 g into peritoneal cavity each time. **b** PCR results of three fragments for each of the three bacteria on agarose gel electrophoresis: NTC: No Template Control, S1: Target fragment 1 of *Mycobacterium smegmatis* which is 322 bp; S2: Target fragment 2 of *Mycobacterium smegmatis* which is 308 bp; S3: Target fragment 3 of *Mycobacterium smegmatis* which is 148 bp; G1: Target fragment 1 of *Mycobacterium goodii* which is 229 bp; G2: Target fragment 2 of *Mycobacterium goodii* which is 840 bp; G3: Target fragment 3 of *Mycobacterium goodii* which is 150 bp; A1: Target fragment 1 of *Mycobacterium abscessus* which is 1061 bp; A2: Target fragment 2 of *Mycobacterium abscessus* which is 320 bp; A3: Target fragment 3 of *Mycobacterium abscessus* which is 133 bp

A 70-year-old female with end-stage renal disease (ESRD) caused by diabetic nephropathy had been treated with PD for 19 months, she underwent temporary hemodialysis and PD protocol adjustments three times due to edema of both lower limbs caused by volume overload because the daily total ultrafiltration volume was about − 200 mL to − 400 mL and the daily water intake was not strictly limited (the specific amount was not recorded), with no residual urine output during that period. She presented to our hospital on February 28, 2019, complaining of abdominal pain. At first, the abdominal pain could be relieved after PD, however, after 1 month, the PD could not alleviate the pain. On admission, her body temperature was 38.4 °C, pulse and blood pressure were normal without any antibiotics or corticosteroids. Physical examinations revealed a soft abdomen with tenderness and rebound tenderness, indicating peritonitis. The skin at the exit-site of the PD catheter and the subcutaneous cuff were without crust formation or erythema, painless, not swollen, and no purulent discharge was observed. Laboratory analysis revealed that the whole blood showed a white blood cell count (WBCC) of 10.17 × 10^9^/L with 89.1% segmented neutrophils, hemoglobin of 7.7 g/dL, and a platelet count of 373 × 10^9^/L. The level of C-reactive protein (CRP) was 211.2 mg/L, procalcitonin (PCT) was 2.940 ng/mL, albumin was 19.2 g/L, blood urea nitrogen was 10.15 mmol/L, and creatinine was 544 μmol/L. The dialysate was turbid, for which the WBCC was 967 × 10^6^/μL with 82.3% neutrophils (Fig. 1a). Therefore, the physician suspected PDAP clinically, which was thought to be a transcatheter infection, because there were no signs of PD catheter exit-site infections (ESI) or tunnel infections (TI). Empirical therapy was performed with intravenous levofloxacin and continuous ambulatory peritoneal dialysis (CAPD), which included cefotiam with intermittent intraperitoneal vancomycin. After 4 days, cultures of dialysate revealed the presence of gram-positive bacilli, which were unidentified to the species level in successive cultures. This supported the diagnosis of PDAP, although the causative organism was unknown. The patient still complained of severe, persistent abdominal pain, with a temperature of 37.4 °C, whole blood WBCC was 9.94 × 10^9^/L with 85.2% segmented neutrophils, the level of CRP was 124.9 mg/L, PCT was 2.260 ng/mL, and dialysate that was cloudy and yellowish, with a cell count of 1444 × 10^6^/L with 83.6% neutrophils. All of these were indicative of a persistent infection. Because conventional cultures demonstrated unidentifiable and fast-growing gram-positive bacilli, the time of culture was prolonged by the laboratory, which tested the minimal inhibitory concentration (MIC) to identify the sensitivity of the pathogenic bacteria. There were no antimicrobial breakpoint criteria for this bacterium; only MIC values or antibacterial ring diameters were reported. The results of MIC for aztreonam, cefixime, cefotaxime, ceftriaone, clindamycin, erythromycin, pristinamycin, teicoplanin, and vancomycin were less than 20 μg/mL, which could help the physician select antibiotics to some extent. Therefore, the therapy was switched to intravenous moxifloxacin, and cefotiam and amikacin were combined intraperitoneally on day 6. The abdominal pain has relieved on day 16, during the dynamic review of ascites tests; inflammatory indicators gradually returned to normal (Fig. 1a), indicating the peritonitis was under control. However, the family and the patient herself refused to remove the peritoneal dialysis tube. Because of the secondary nosocomial infections, including oral fungal and lung infections, the peripheral blood inflammation indicators increased again, and she was discharged on day 22, March 21, 2019, and died a few days later.

On March 26, we received the results of a bacterial culture in dialysate from laboratory identified as *M. smegmatis,* collected on day 6 without sensitivities results. According to clinical experience and the literature, we concluded that *M. smegmatis* is rarely pathogenic; therefore, further tests were performed to determine the pathogen. To verify the growing bacteria in this patient, the stored sample, coded R1904155ADPN257, was retested using mNGS with permission from the family of the patient. The results identified *M. smegmatis* (24,708 reads), *M. abscessus* (9224 reads), *M. goodii* (8305 reads), and three sequence fragments from each of these species were compared with those available in GenBank sequence database respectively. All shared a high degree of homology with the bacterial sequences in GenBank database. The three sequence fragments most closely matching *M. smegmatis* in the NCBI database were all *M. smegmatis*, including one identified as *M. smegmatis* MKD8 (GenBank accession number CP027541.1) and the other two as *M. smegmatis* genome assembly NCTC8159 (GenBank accession number LN831039.1). The three sequence fragments most closely matching *M. abscessus* in the NCBI database were all *M. abscessus* FLAC046 (GenBank accession number CP016192.1). The three sequence fragments that most closely matching *M. goodii* in the NCBI database were all *M. goodii* X7B (GenBank accession number CP012150.1). Polymerase chain reaction (PCR) confirmed that the three bacteria were present in the specimen, and their cycle threshold values were 16, 18, and 15.5, respectively. These results were different from those of routine cultures, especially that of *M. abscessus*, which were suspect bacterial species and pathogenic bacteria in the dialysis effluent. There were very high sequence similarities in the different species of NTM. In order to validate mNGS results, we performed first whole-genome sequences on R1904155ADPN257, three primer pairs were also used to specifically amplify the target fragments (Table 1) and the PCR results are shown in Fig. 1b. The above fragments were aligned separately in the NCBI database, and the final results demonstrated that three species, *M. smegmatis, M. abscessus,* and *M. goodii*, coexisted simultaneously in the specimen.

**Table 1:** Results of three primer pairs used to specifically amplify target fragments

Primer	Sequence(5′ → 3′)	Target fragment (bp)	Species
MS-2F1	GGTTCCCGAACTGAACTGCC	322	*Mycobacterium smegmatis*
MS-2R1	CACCACGGTCAAGACGATGAA		
MS-2F2	ATGCGGAAGACGACTGGG	308	
MS-2R2	TGGGTGCTTACTGACTTGAATG		
MS-F1	TACAGGCCTTTCGAGGATCC	148	
MS-R1	GGTACTCCGAACGATGATCCT		
MG-2F1	GTGCCATGTCCTACCTGAAGAG	229	*Mycobacterium goodi*
MG-2R1	TGGTCGGGCTTGCTCCTA		
MG-2F2	GGAACGACGCAACGGTAGA	840	
MG-2R2	CACTGATGGACGCCTTGAGTAA		
MG-F1	AGTTCATCATGGTCGATCGC	150	
MG-R1	CATGACCAAGCCGTTCCAC		
MA-2F1	GCCGACGAGACCAACATCC	1061	*Mycobacteroides abscessus*
MA-2R1	CAGTGCTCACCCTTGCCAGTA		
MA-2F2	CGAATCCACCACGATGACG	320	
MA-2R2	GTCGAGGACGATCTTGAGCC		
MA-F1	GTTGCGATCTAGGTGTGCTG	133	
MA-R1	TCAAAGTACCGCTCAATCGC		

Thus, we confirmed that the patient was afflicted with a mixed infection of PDAP caused by multiple NTM. We did not suspect that *M. smegmatis* was responsible for the peritonitis.

## Discussion and conclusions

The patient was treated with PD, and was in a special immunosuppressive state and complained of abdominal pain with low fever. The examination indicated a soft, tenderness abdomen with rebound tenderness, the inflammatory indicators of whole blood were elevated, the dialysate was cloudy and yellowish, in which the WBCC was high and neutrophils were predominant. Routine cultures revealed gram-positive bacilli. According to PDAP diagnostic criteria [20], we diagnosed the patient with PDAP. Since she had no history of tuberculosis (TB); the signs of active pulmonary TB, alveolar infiltration, cavitation, and lymphadenopathy were not observed in the chest computed tomography [21]. *M. smegmatis* was isolated by conventional culture, but mNGS and first whole-genome sequences both identified three species in the dialysate, including *M. smegmatis, M. abscessus,* and *M. goodii*. This is the first case with a firm evidence of PDAP, where conventional tests isolated a rare NTM with weak pathogenicity. A variety of pathogens may coexist, their detection largely relies on the development of methods.

A typical characteristic of this case was the presence of three distinct NTM species, and the pathogenic bacterium was not clear. It is well-known that NTM are a group of environmental organisms, and their identification does not indicate the presence of NTM diseases [22]. They commonly cause skin and soft tissue, bone, and respiratory infections, especially in the people with immunosuppression, exposure to broad-spectrum antibacterial drugs, and inadequate dialysis and insufficient residual kidney function [23]. Various rapidly growing mycobacteria (RGM) species are associated with catheter infections, which appear to be caused by prolonged retention of catheters, such as central venous catheters, abdominal catheters, and shunt catheters. *M. smegmatis* groups (including *M. smegmatis*, *M. goodii,* and *M. wolinskyi*) are not the main species responsible for this type of infection [24–26]. As opportunistic pathogens in NTM, *M. smegmitis* and *M. goodii* have lower incidences than *M. abscessus*, only appearing in case reports [27, 28]. *M. smegmitis* was first isolated by Lustgarten in 1885 and was recognized as a human pathogen by Vonmoos et al. in 1986 [29]. Fewer than 50 cases have been reported, and it is worth mentioning there has been only one case report about PDAP that identified *M. smegmatis*, which used 16S-RNA sequencing to identify the bacterium [8]. In *M. smegmatis*-induced catheter-related infections, in addition to the manifestations of fever and discharge of pus, patients may suffer from immunosuppression [24, 25, 30]. However, others suggest that *M. smegmatis* rarely causes catheter-associated infections regardless of the immune status of the patient [31]. The relationship between *M. smegmatis* and pathogenesis is still debated. Some believe that *M. smegmatis* should be considered a pathogenic bacterium in chronic cutaneous and soft tissue infections, with a history of soil-contaminated wounds and use of glucocorticoids [27]. However, as early as 1979, it was believed that *M. smegmatis* was not pathogenic to humans [32], and it colonized the environment. Despite repeated cases, clinicians are cautious about its clinical significance.

The previous case reports on *M. smegmatis* identification methods include bacterial culture [33, 34], and molecular methods, such as PCR restriction fragment analysis [33], sequencing specific genes, *hsp*6*5* [35] and 16S–23S rRNA gene sequencing [33], matrix-assisted laser desorption/ionization-time-of-flight mass spectrometry (MALDI-TOF MS) [33, 36], and 16S rRNA are the most commonly used [28, 33]. In previous studies, the tests could only show the presence of *M. smegmatis* in the specimens and did not have the ability to verify mixed infections, which could not be realized by mNGS until now [37]. To the best of our knowledge, this is the first report of a mixed NTM infection based on mNGS after the routine culture isolated *M. smegmatis*. We suspect that *M. smegmatis*, the result of the conventional bacterial culture, was not the causative pathogen. Based on our findings, it is reasonable to suspect that the real pathogen was not *M. smegmatis,* which was thought to cause infections in previous case reports, whereas other microbes with lower abundance but greater pathogenicity could not be identified because of the limitations of the detection technologies. Currently, It is possible for this supposition to be verified by future similar cases or by prior similar cases with preserved specimens. Thus, when a patient is suspected of clinical infection, the results of conventional tests might be inconsistent with manifestations, only microbes with weak pathogenicity isolated, or the results of the etiology were negative, gene sequencing could be the best choice. It was the application of mNGS, which has the best results, that revealed the instance of a mixed infection in this case.

*M. goodii*, a new RGM that was differentiated from *M. smegmatis groups* in 1999 by Brown et al., has rarely been identified as a pathogen [38] and is currently considered as a zoonotic pathogen [39]; it is associated with post-traumatic wound infections; there are only case reports on surgical sites, repair materials (e.g., pacemaker implant, prosthetic joint, hernia sac mesh, etc.), bone infections, and pulmonary diseases [40]; a considerable part of these involve iatrogenic infections, which bring great challenges to clinicians [41, 42]. However, to our knowledge, this case was the first time that *M. goodii* was identified in PDAP. As we have said before, the existence of microbes does not mean infection, except in very rare conditions [22], and most guidelines still regard *M. goodii* as a commensal bacterium with little clinical significance [38]. Therefore, it is unclear what role *M. goodii* played in this case, and we prefer to think it is a commensal bacterium without pathogenicity.

In contrast to *M. smegmatis* and *M. goodii, M. abscessus* is a multidrug-resistant NTM responsible for a wide spectrum of skin and soft tissues infections [43, 44] and pulmonary disease, especially in vulnerable hosts with underlying structural lung disease [22] and other infections [45]. Under the new diagnostic criteria, this was revealed as the most frequently encountered RGM in human infections [46]. As for PDAP caused by NTM, *M. abscessus* also plays an important role. A review in Taiwan indicated that RGM represented the major etiology of abdominal NTM infections, especially *M. abscessus* [47], although it most often appeared in case reports [6, 48]. In addition, *M. abscessus* is naturally resistant to traditional first-line anti-tuberculosis drugs and carries a gene erm that can induce clarithromycin resistance [49], which makes treating it is a challenge. Unlike other opportunistic pathogenic bacteria, *M. abscessus* can cause disseminated infections in immunocompromised hosts, and bacteremia can occur in the context of dialysis catheters [50, 51]. *M. abscessus* is considered the most pathogenic antibiotic-resistant mycobacterium [52, 53]. Thus, we conclude that *M. abscessus* is the causative pathogen instead of *M. smegmatis,* which was previously isolated by conventional methods.

It is reasonable to conclude that in patients with PDAP, even if conventional tests isolate *M. smegmatis*, there could be other species in NTM with lower abundance but greater pathogenicity which are more likely pathogens. When a bacterium with weak pathogenicity, such as *M. smegmatis*, or is inconsistent with clinical manifestations is isolated by conventional methods in a patient who is suspected of infection, clinicians should be vigilant and conduct additional tests to determine the presence of rare or even newly discovered microbes, which is indicated by mixed infections. Thus, when *M. smegmatis* has been identified in clinical specimens and thought to be responsible for the infections in the published case reports, the pathogenic bacteria might not have been identified. If mNGS could be performed on these specimens, there might be different findings, which could support our supposition. With the development and extensive applications of detection techniques, the emergence of similar cases, and the attention of clinicians, this supposition could be more strongly supported. This is the first case of the coexistence of multiple NTM detected in PDAP. This case indirectly demonstrated the pathogenicity of *M. abscessus*, and mNGS was valuable in the identification of the coexistence of multiple bacteria.

## Supplementary Information

**Additional file 1.** mNGS and first whole-genome sequences. The sequencing results and sequence alignment of specific sequence target amplified fragments.

**Additional file 2.** PCR results of three fragments for each of the three bacteria on agarose gel electrophoresis which is the original, uncropped.

Additional file 3. CARE Checklist of information to include when writing a case report

## Data Availability

All data generated or analyzed during this study are included in this published article and its supplementary information files.

## Abbreviations

PDAP: Peritoneal dialysis-associated peritonitis
PD: Peritoneal dialysis
NTM: Nontuberculous mycobacteria
MTB: *Mycobacterium tuberculosis*
MAC: *Mycobacterium avium* complex
MABC: *Mycobacterium abscessus* complex
ESRD: End-stage renal disease
WBCC: White blood cell count
CRP: C-reactive protein
PCT: Procalcitonin
ESI: Exit-site infection
TI: Tunnel infection
CAPD: Continuous ambulatory peritoneal dialysis
MIC: Minimal inhibitory concentration
TB: Tuberculosis
RGM: Rapidly growing mycobacteria
