# Supplementary figures and images for: Mixed infection of three nontuberculous mycobacteria species identified by metagenomic next-generation sequencing in a patient with peritoneal dialysis-associated peritonitis: a rare case report and literature review
Source: BMC Nephrol. 2023 Apr 13;24:95. doi: 10.1186/s12882-023-03156-8 (PMC10099677; doi:10.1186/s12882-023-03156-8)

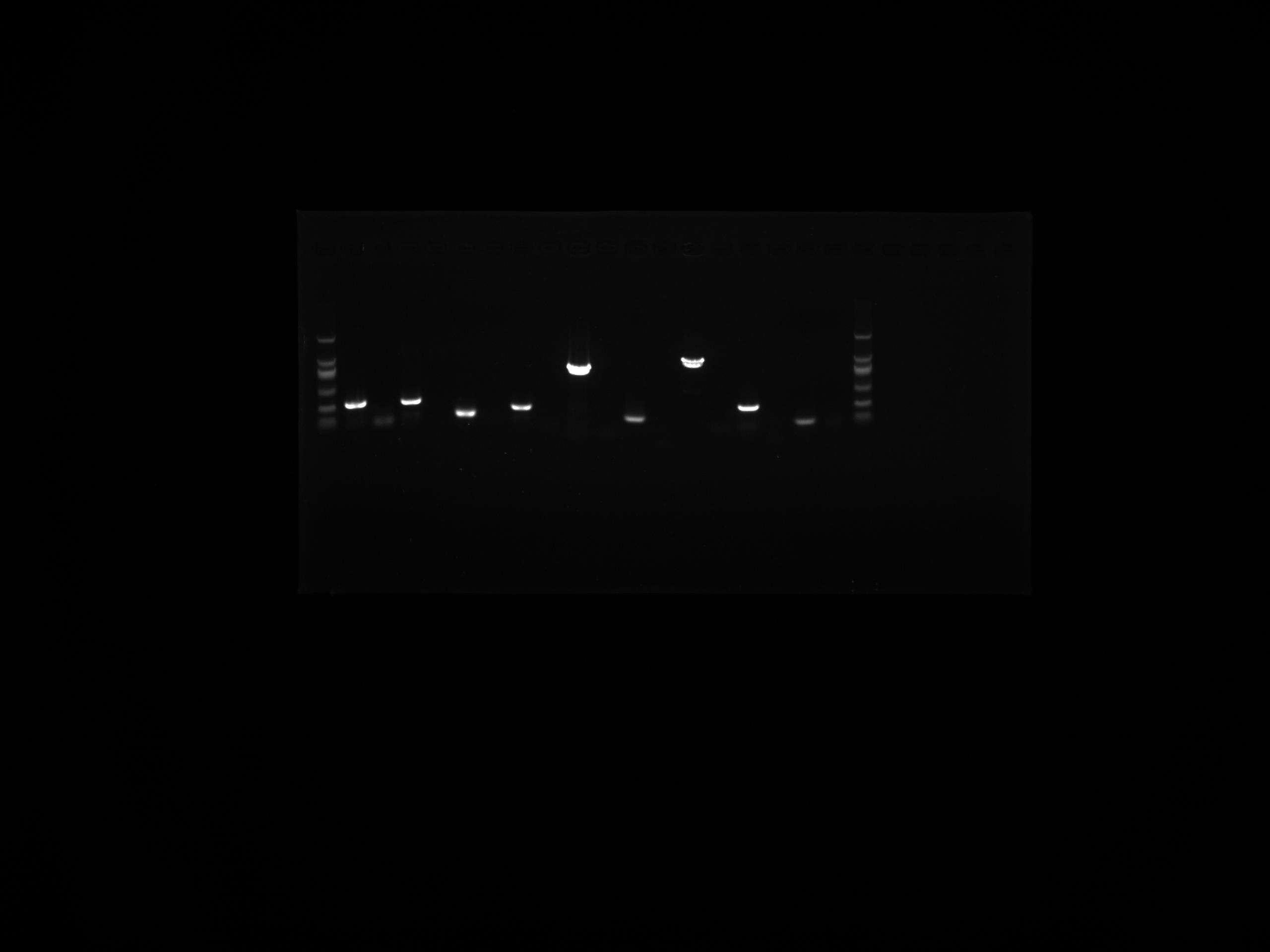

Supplement: Supplementary file 2 — Additional file 2. PCR results of three fragments for each of the three bacteria on agarose gel electrophoresis which is the original, uncropped. [file 12882_2023_3156_MOESM2_ESM.jpg]
